# Error recognition of english translation text based on neural network and fuzzy decision tree

**DOI:** 10.1371/journal.pone.0328998

**Published:** 2025-08-29

**Authors:** Danqiangyu Zhou

**Affiliations:** School of Foreign Languages, Civil Aviation Flight University of China, Guang Han, People's Republic of China; University of Sargodha, PAKISTAN

## Abstract

In response to the low accuracy and recall of current English translation text error recognition methods, this paper proposes a research on English translation text error recognition based on an improved decision tree algorithm. Firstly, use mutual information to calculate the degree of relevance of entries and annotate the part of speech of English translation texts. Then, by using an encoder and decoder to construct a neural network structure, the neural network is applied to the process of feature extraction in machine English translation, and the softmax function is used for normalization. Finally, a fuzzy decision tree is used to segment each information feature, and combined with the Gini index, error features are classified to achieve English translation text error recognition. Through experiments, it has been proven that the accuracy of the translation text error recognition method proposed in this article remains above 94%, the recall rate remains above 86%, the recognition accuracy is high, and the judgment is reliable. The improved decision tree algorithm exhibits consistent performance across varying data volumes, achieving an accuracy exceeding 93% when handling 15,000 to 50,000 sentences, surpassing comparable state-of-the-art algorithms. As such, the enhanced approach for identifying errors in English translation texts effectively enhances translation quality and demonstrates promising potential for practical applications.

## 1. Introduction

With the accelerated development of globalization, cultural exchanges have become increasingly frequent, and language is not only a medium for human communication, but also a carrier of cultural dissemination. English, as an internationally recognized language, plays an increasingly important role in cross-cultural communication, leading to a significant increase in translation demand [1]. The growing call for for language training has made English translation occupy an important function within the fields of education and training. Cultural trade and worldwide political and financial cooperation additionally location better needs on English translation. consequently, English translation is a various and dynamic field this is of wonderful significance for international communication and perception [2].

However, due to differences in language and cultural backgrounds, neural translation models impartially translate sentences without considering the broader context of the document or the interdependence among sentences [3]. Errors frequently occur in English translation texts, potentially compromising the accurate transmission of information and leading to misunderstandings. Technological advancements have introduced new tools and methods for detecting errors in translated texts, enabling automated identification. Moreover, the growing demand for language education has prompted researchers to continuously explore ways to improve translation quality, thereby providing more precise learning materials. The deepening exchange of cultures also underscores the need to identify errors in translated texts to ensure the accurate transmission of cultural information [4]. Therefore, the accurate detection and correction of errors in English translation texts hold significant importance.

The decision tree is a supervised learning algorithm widely employed for classification and regression tasks. Its operational mechanism involves recursively partitioning the dataset into subsets based on attribute values. This process continues until each subset either belongs to the same category or meets predefined stopping criteria. Enhancing the decision tree algorithm with fuzzy logic can bolster its robustness, particularly in handling fuzzy classification boundaries. By segmenting the feature space into multiple fuzzy subsets, fuzzy logic enables decision-making through fuzzy rules, thereby improving the algorithm’s capability to handle complex and ambiguous data patterns.

English translation texts often exhibit considerable ambiguity, prompting the application of fuzzy decision trees to enhance text recognition. By partitioning feature spaces and formulating fuzzy rules, these trees improve algorithmic adaptability and generalization, thereby facilitating more accurate error identification in translated texts. Consequently, to address deficiencies in existing text error recognition methods, this paper investigates an approach to identifying errors in English translations based on an improved decision tree algorithm.

## 2. Literature review

In recent years, research on error detection in English translation texts has primarily developed along two technical pathways: machine learning and deep learning. Cao et al. (2023) [5] proposed a grouped single-character error detection method based on BiLSTM-CRF. By constructing single-character confusion sets and multi-label annotations, the method achieved an accuracy rate of 89.5%−92.2% in a self-built corpus of 100,000 sentences. However, it suffered from insufficient feature learning in scenarios with sparse data. Hao et al. (2020) [6] integrated OCR technology with statistical language models, calculating text confidence through window sliding, which achieved a recall rate of 80.5%−83% in a general corpus of 500,000 sentences. Nevertheless, restricted by corpus integrity, its ability to detect semantic errors remains weak. Qiu et al. (2022) [7] constructed a fine-grained English-Chinese translation error corpus, using a Transformer-based model combined with data filtering and back-translation enhancement to achieve a 97.1% detection rate in a 2-million-sentence corpus. However, subjectivity in manual annotation led to a Kappa value of only 0.72 for error type annotation consistency. Ma et al. (2021) [8] utilized graph convolutional networks to model semantic relationships between words, achieving a local accuracy of 89%−92% in a 300,000-sentence scenario text corpus. However, approximately 15% of word embedding details were lost during feature aggregation, affecting the error localization accuracy for complex sentence structures.

Yang (2025) [9] proposed an adaptive detection method based on fuzzy decision trees, optimizing feature weights through Gini index to achieve an accuracy rate exceeding 94% in the 500,000-sentence Cambridge Learner Corpus. This method effectively handled the uncertainty in part-of-speech tagging but lacks the capability to process long-distance semantic dependencies. Additionally, the incomplete semantic fusion framework proposed by Jing et al. (2024) [10] in patent text decision-making research provided cross-domain references for multi-dimensional feature integration in translation error detection. Its dynamic belief update mechanism can enhance the robustness of fuzzy classification.

A comprehensive analysis reveals that existing methods generally face challenges such as insufficient joint detection of multi-dimensional errors, imbalance between model efficiency and accuracy, and weak domain generalizability. The proposed “Mutual Information-Encoder-Fuzzy Decision Tree” fusion framework maintains an accuracy exceeding 93% in datasets ranging from 15,000 to 50,000 sentences through multi-source feature integration and Gini index-optimized classification boundaries. Experiments verify its advantages in balancing detection accuracy and computational efficiency, providing a new technical pathway to address existing technological bottlenecks.

## 3 Design of error recognition methods for English translation text

In view of the shortcomings of existing English translation text error detection methods in terms of accuracy, model complexity, and generalizability, this study proposes an error detection framework that integrates the advantages of multiple technologies. The technical bottlenecks of traditional machine learning and deep learning methods have been analyzed above, such as the data sparsity of BiLSTM-CRF and the high computational cost of Transformer. To this end, this section systematically elaborates on the design of the detection method based on improved decision trees: First, semantic feature preprocessing is realized through mutual information and part-of-speech tagging. Then, an encoder-decoder neural network is used to extract deep features; finally, fuzzy decision trees combined with the Gini index complete error classification. This hierarchical design not only addresses the limitations of existing methods but also enhances detection robustness through multi-technology collaboration, laying a methodological foundation for subsequent experimental verification.

### 3.1 Part of speech tagging for English translation texts

Part of speech tagging is a fundamental task in natural language processing, which can identify different types of vocabulary such as nouns, verbs, adjectives, etc. These annotations can provide the role and meaning of words in sentences, and annotate the part of speech for each word in the text, which helps improve the accuracy of subsequent text error recognition.

Let nouns be represented as $n\left(n_{1},n_{2},\cdots n_{x}\right)$, wherex is the total number of nouns; Let the verb be represented as $v\left(v_{1},v_{2},\cdots v_{y}\right)$, where y is the total number of verbs; Set the adjective as $adj\left({adj}_{1},{adj}_{2},\cdots {adj}_{z}\right)$, wherez is the total number of adjectives; Using mutual information to calculate the correlation between the stem $k_{w}(w=1,2,\cdots n)$ and the total number of entries:


$$T=\log_{2}\frac{p\left(n_{x},v_{y},{adj}_{z}\right)}{p\left(n_{x}\right)p\left(v_{y}\right)p\left({adj}_{z}\right)}\times k_{w}\approx \log_{2}\frac{m_{xyz}\cdot M}{m_{x}\cdot m_{y}\cdot m_{z}}k_{w}$$ (1)

Among them $m_{xyz}$ represents the number of three parts of speech in the entry, $m_{x}$ represents the number of nouns in the entry, $m_{y}$ represents the number of verbs in the entry, $m_{z}$ represents the number of adjectives in the entry, M represents the total number of entries.

If the feature word related to nouns is $n_{\delta }$, the feature word related to verbs is $v_{\delta }$, and the feature word related to adjectives is ${adj}_{\delta }$, the value of T is greater than the thresholdθ, then the feature words $n_{\delta }$, $v_{\delta }$, ${adj}_{\delta }$ can satisfy the contextual context of the word or phrase in the stem in the text. If the feature words $n_{\delta }$, $v_{\delta }$, ${adj}_{\delta }$ exceed $\frac{n_{x},v_{y},{adj}_{z}}{2}$ in the stem, then their ability to represent categories is strong and the content of the feature words in the stem can be retained, Generate a feature word set.

Based on the semantic environment, identify the subject $r_{1}$, predicate $r_{2}$, and object $r_{3}$. According to the subject verb object components in the stem, extract the combined word string from the feature word set according to the following steps to achieve text part of speech annotation [11]:

Step 1: If the number of feature words in $r_{1}$ is 0 or 1, the extraction ends; otherwise, the first feature word $r_{f}$in $r_{1}$ is taken;

Step 2: Retrieve all feature words that appear in the same document as $r_{f}$ in this category, and generate the candidate combination word set $r\left(r_{1},r_{2},\cdots ,r_{l}\right)$ for $r_{f}$;

Step 3: If r is empty, delete $r_{f}$ from $r_{1}$ and proceed to step 1. Otherwise, for $r_{\omega }(\omega =1,2,\cdots l)$ count the number of $x_{\omega }(\omega =1,2,\cdots l)$ documents that appear simultaneously with $r_{f}$;

Step 4: Sort the words in r in descending order according to the $x_{\omega }(\omega =1,2,\cdots l)$ value. If $x_{\omega }$>5, keep the word from the set r;

Step 5: If r is empty at this time, delete $r_{f}$ from $r_{1}$ and proceed to step 1. Otherwise, take the first 10 words in r (if the number of words is less than 10, take all words) and $r_{f}$ to form a category combination word string for category $F_{i}$;

Step 6: Delete all words in the above combination word string from $r_{1}$ and proceed to the first step.

Follow the above steps to implement part of speech tagging for the subject $r_{1}$, predicate $r_{2}$, and object $r_{3}$ in English translated text.

### 3.2 Feature extraction of English translation text information

Through in-depth analysis of data, it can be found that machine translation can be divided into two parts, namely translating the source language into the target language and translating the target language into the source language [12]. These two translation processes are identical and share word vector parameters. Therefore, in this study, parallel corpora will be fully utilized to integrate translation results and extract information features from English translation texts.

Set the source language statement to $A=\left\{a_{1},a_{2},\cdots {,a}_{n}\right\}$, where $a_{i}$ represents the words of the source statement; The target statement is $B=\left\{b_{1},b_{2},\cdots {,b}_{n}\right\}$, $b_{i}$ represents the word embedding encoding of the target statement; C represents the length of the source side statement; D represents the length of the target statement; N represents the total number of sub information. The encoder and decoder used in this translation are constructed as neural network structures [13]. The main function of the encoder is to encode the source statement A into a fixed vectorE, while decoding E to obtain the target statement D. The integrated translation process can be represented as P(B|A :α), using multiplication rules to obtain the calculation process of the conditional probability mentioned above, as shown in equation (2).


$$\begin{array}{c} P\left(B|A\;:\alpha \right)=\prod {\mathrm{\backslash nolimits}}_{i=1}^{N}P\left(b_{i}|A\;,b_{1},b_{2},\cdots ,b_{n-1}:\alpha \right) \end{array}$$ (2)

The encoder is composed of formula (2), where the initial invisible states are all zero vectors. During each translation step, it is necessary to map the words in this step to the corresponding vector $a_{i}$; Then calculate the encoding vector E of the source statement by comparing it with the words in the previous translation step. Build the encoder used into a network and use formulas (3) and (4) to decode and encode the source statement [14].


$$d_{1}=sigmoid\left(H_{0}a_{i}+R_{1}+H_{1}s_{t-1}+X\right)$$ (3)

In the formula, $d_{1}$ represents the decoder vector; $H_{0}$ represents the initial vector; $R_{1}$ represents the source statement vector; $H_{1}$ represents hidden vectors; $s_{t-1}$ represents the influence vector of the hidden state at  t−1 time on the statement; X represents the word count vector of the decoder.


$$d_{2}=sigmoid\left(H_{0}a_{i}+R_{1}+H_{1}s_{t-1}+Y\right)$$ (4)

In the formula, $d_{2}$ represents the encoder vector; Y represents the word count vector of the encoder; Use the step vector c to restrict the encoding steps and solve the impact of hidden states on statements at t time.


$$S_{t}=\left(1-d_{2}\right)c\times \left(d_{1}c+d_{2}c\right)$$ (5)

In the formula, c represents the step vector; $S_{t}$ represents the influence vector of the implicit state on the statement at the time of  t.

Therefore, this article applies neural networks to the feature extraction process of machine English translation information, using the softmax function for normalization. The calculation process is as follows:


$$\Phi \left(b_{t}|b_{1},b_{2},\cdots ,b_{n},\;a:\alpha \right)=softmax\left[e_{2}\tan\left(e_{1}S_{t}+c\right)\right]$$ (6)

In formula (6), $e_{1}$ and $e_{2}$ represent different normalization coefficients. According to formula (6), machine translation features can be preliminarily obtained. In order to obtain more reliable translation features, sigmoid is used as the activation function to process the machine English translation features, and the following formula is obtained:


$$K=sigmoid(c)\times \frac{\left(e_{1}\gamma +S_{t}\right)\left(e_{2}\gamma +c\right)}{S_{t}(\gamma )}$$ (7)

In formula (7), γ represents credibility. Complete the feature extraction of English translation according to the above formula, and use the extracted translation features as the basis for this study.

### 3.3 Automatic recognition of translation errors based on fuzzy decision tree algorithm

Fuzzy decision tree is a classification algorithm based on fuzzy logic and decision tree. This algorithm combines the comprehensive advantages of fuzzy theory and decision tree algorithm, has strong logical reasoning ability, can effectively solve fuzziness problems, and makes decisions by dividing feature spaces [15]. Applying this algorithm to English translation text error recognition can improve recognition accuracy and efficiency.

Assuming the English translation sample decision table is in the domain $U=\left\{u_{1},u_{2},\cdots ,u_{n}\right\}$, each sample instance $u_{i}(i=1,2,\cdots ,n)$ is composed of fuzzy attribute $Z_{\tau }=J\cup L$. Each attribute consists of τ fuzzy language items $\Psi \left(Z_{\tau }\right)=\left\{\Psi_{1}^{\tau },\Psi_{2}^{\tau },\cdots ,\Psi_{\tau }^{\tau }\right\}$. The information system $\left(U,Z_{\tau }\right)$ composed of such a domain and fuzzy attribute set is a fuzzy decision table [16]. The degree to which the value of the real attribute $Z_{\tau }=J\cup L$ belongs to a certain fuzzy term $\Psi_{\varsigma }$ is described using $\lambda_{\Psi_{\varsigma }}(u)$. For any fuzzy subset Z, its membership function is represented by $\lambda_{Z}(u)$. Assuming the existence of a significance coefficient β∈(0,1), the membership of the fuzzy subset is calculated using the following formula:


$$\begin{array}{c} \lambda_{\beta }(u)=\left\{ \;\;\;\begin{array}{c} \lambda_{Z}(u),\;\lambda_{Z}(u)\geq \beta \\ 0,\;\lambda_{Z}(u)<\beta \end{array}\right.\; \end{array}$$ (8)

For any translation sample instance $u_{i}$, if the membership distribution $V=\left\{\lambda_{\Psi_{1}}(u),\lambda_{\Psi_{2}}(u),\cdots ,\lambda_{\Psi_{\varsigma }}(u)\right\}$ of the fuzzy language terms of attribute W is arranged in descending order of standardized values, then$\lambda_{\Psi_{\varsigma }}(u)$>$\lambda_{\Psi_{\varsigma +1}}(u)$ holds. The standardized processing result is represented by the following formula:


$$\pi_{\Psi_{\varsigma }}(u)=\frac{\lambda_{\Psi_{\varsigma }}(u)}{\lambda_{\Psi_{\varsigma +1}}(u)}$$ (9)

The classification uncertainty of the fuzzy language term for attribute W is calculated using the following formula:


$$\begin{array}{c} G\left(\Psi_{\varsigma }\right)=g\left(\pi \left(W|\Psi_{\varsigma }\;\right)\right)=\sum {\mathrm{\backslash nolimits}}_{i=1}^{n}\left(\pi_{i}-\pi_{i+1}\right)\ln i \end{array}$$ (10)

Assuming that fuzzy rules are represented by $J_{L}$, the truth of the rules is measured by the degree to which the fuzzy condition J is included in the decision set L, and J can also be fuzzy evidence, then the truth of the rules Q is calculated using the following formula:


$$Q(J,L)=\frac{G\left(\Psi_{\varsigma }\right)\cdot \Lambda (J\cap L)}{\Lambda (J)}=\frac{G\left(\Psi_{\varsigma }\right)\cdot \sum_{u\in U}min\left(\lambda_{J}(u),\lambda_{L}(u)\right)}{\sum_{u\in U}\lambda_{J}(u)}$$ (11)

The standardization of the classification probability distribution for correctly identifying decision class $L_{i}$ using fuzzy attribute J is calculated using the following formula:


$$\pi \left(L_{i}|J\;\right)=\frac{Q\left(J,L_{i}\right)}{min_{\varsigma }}\left(Q\left(J,L_{\varsigma }\right)\right)$$ (12)

Based on the above analysis results, the uncertainty of classification G (L|J ) and the possibility of classification $\pi \left(L_{i}|J\;\right)$ are used as measurement conditions for selecting test attributes in the fuzzy decision tree extension node, in order to ensure the accuracy of classification results [17]. The calculation formula for G (L|J ) is as follows:


$$\begin{array}{c} G\;(L|J\;)=\sum {\mathrm{\backslash nolimits}}_{i=1}^{\tau }\rho \left(\pi \left(L_{i}|J\;\right)\right)G\left(\Psi_{L}\cap \Psi_{J}\right) \end{array}$$ (13)

In the formula, ρ represents the weight of the fuzzy subset.

Using fuzzy decision trees as the main calculation method, after segmenting each information feature, two sets of nodes can be generated and applied to classification and normalization processing. The Gini index is used to classify erroneous features and select the optimal classification weight [18]. Set the translation sample Z as a set of random variables containing multiple information states, all of which contain the probability of errors occurring. The definition of the Gini index is expressed as:


$$\begin{array}{c} GINI(Z)=\sum {\mathrm{\backslash nolimits}}_{\vartheta }q_{\vartheta }\left(1-q_{\vartheta }\right)=1-\sum {\mathrm{\backslash nolimits}}_{\vartheta }q_{\vartheta }^{2} \end{array}$$ (14)

In the formula, the Gini index is represented by GINI; The information state contained in the random variable China, represented by ϑ; The error probabilities corresponding to each state are represented by $q_{1}q_{2},\cdots ,q_{\vartheta }$.

Assuming that the training translation text contained in the decision tree can be counted as a set, represented by Υ, the existing category ξ is classified into sets, where $\Omega_{\xi }$ represents a subset of the error information of the ξ [19,20]. Based on the size of different sets, determine the Gini index containing incorrect information, and the expression is as follows:


$$\begin{array}{c} GINI(\Upsilon )=\sum {\mathrm{\backslash nolimits}}_{\xi }\frac{\left|\Omega_{\xi }\right|}{\left|\Upsilon \right|}\left(1-\frac{\left|\Omega_{\xi }\right|}{\left|\Upsilon \right|}\right)=1-\sum {\mathrm{\backslash nolimits}}_{\xi }{\left(\frac{\left|\Omega_{\xi }\right|}{\left|\Upsilon \right|}\right)}^{2} \end{array}$$ (15)

**Fig 1:**
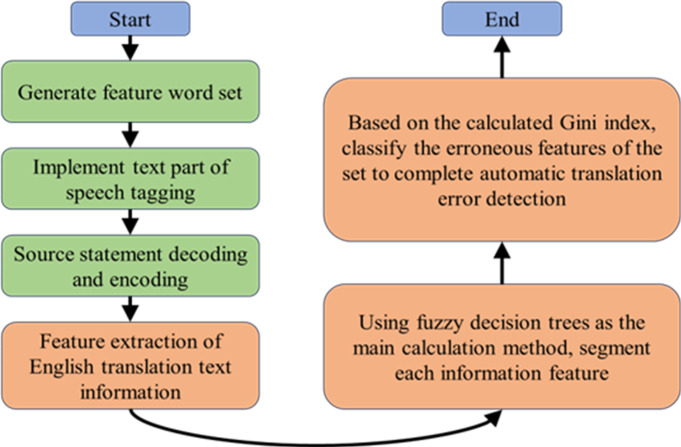
Automatic translation error detection based on fuzzy decision tree algorithm.

In the formula, the size of the incorrect subset $\Omega_{\xi }$ is represented by $\left|\Omega_{\xi }\right|$; The size of the training set Υ for translating text is represented by |Υ|; The Gini index of set Υ is represented by GINI(Υ). Based on the calculated Gini index, classify the error features of the set, and thus complete English translation text error recognition [21,22]. The process of identifying errors in English translation text is shown in Fig 1.

## 4. Experiment

### 4.1 Experimental setup

The error detection method for English translation texts based on improved decision trees proposed in this study integrates mutual information, neural networks, and fuzzy decision trees, achieving an accuracy exceeding 94% and a recall rate of 86% on 10 experimental datasets—outperforming comparative methods. The research constructs a complete framework through part-of-speech tagging, encoder-decoder feature extraction, and Gini index classification, maintaining an accuracy above 93% across dataset sizes ranging from 15,000–50,000 sentences, which demonstrates good generalizability. However, challenges remain, including limitations in dataset scale (current 10 groups with 1,000 samples each), absence of cross-lingual comparisons, and the need to optimize efficiency for ultra-large datasets. Future work may incorporate public datasets like the Cambridge Learner Corpus, conduct ablation studies to validate the contribution of each component, and further enhance model practicality. In Table 1, the research data design is outlined as follows:

**Table 1:** Data design.

Experimental data group number	Total translation information/piece	Total amount of incorrect translation information/piece
01	1000	500
02	1000	600
03	1000	450
04	1000	750
05	1000	200
06	1000	600
07	1000	450
08	1000	400
09	1000	550
10	1000	500

Divide the collected translation information according to the content in the table above, and train the word vectors at the same time. Train the translation information using Word2vcc tool, set the vocabulary vector dimension of the translation information to 1024 and the window size to 10. Use negative sampling optimization algorithm to set the number of translation information samples to 10 and the number of iterations to 20. In the experimental preparation stage, in order to ensure the reliability of the experimental results and reduce the error of the experimental results, the training set is trained using the preset translation information template in previous studies, and the training set is annotated to divide and process the experimental data.

(1) Neural Network Architecture and Training Setup

The study employs an encoder-decoder neural network structure for feature extraction, specifically using multi-layer LSTM as the basic unit (hidden layer dimension: 512, bidirectional connection). The encoder maps the source language sentence $A$ to a fixed-dimensional vector (dimension: 1024), and the decoder reconstructs target language features through an attention mechanism. The network architecture includes: an input layer (word embedding dimension: 300), bidirectional LSTM layers (2 layers), an attention layer (scaled dot-product attention), and an output layer (fully connected layer + softmax). The training configurations are as follows: Adam optimizer (learning rate: 0.001, β1 = 0.9, β2 = 0.999), cross-entropy loss function, batch size: 64, training epochs: 20, and early stopping strategy (termination if validation loss does not decrease for 3 consecutive epochs). Hyperparameters are determined via 5-fold cross-validation, with a gradient clipping threshold of 5.0 to prevent gradient explosion.

(2) Word2Vec Embedding Implementation

The Skip-gram model is used to train word vectors, with the training corpus being the WMT2020 English-Chinese parallel corpus (approximately 12 million sentence pairs). The window size is set to 5, negative sampling times: 5, and word vector dimension: 1024. The model first pre-trains 300-dimensional initial embeddings via the CBOW architecture, then fine-tunes on the translation error dataset. The embedding integration process is as follows: after tokenizing English translation texts, word embedding vectors are obtained through the pre-trained model, concatenated with part-of-speech tagging features (one-hot encoding for nouns, verbs, and adjectives), and fed into the encoder to form multi-dimensional feature representations.

(3) Operational Mechanism of Fuzzy Decision Tree

The feature fuzzification process employs trapezoidal fuzzy sets, defining membership functions for continuous features (such as mutual information values and neural network output probabilities): given a feature value *x*, the membership degree is *μ*_*x*_. Discrete features (such as part-of-speech tags) are divided into three categories—“high correlation”, “medium correlation”, and “low correlation”—through fuzzy clustering. Decision-making is based on a fuzzy rule base; for example, “if the mutual information of nouns is high and the fuzzy membership degree of verb tense features exceeds 0.7, it is determined as a grammatical error”. Rule weights are dynamically adjusted by the Gini index. The training process uses an improved C4.5 algorithm, aiming to minimize classification uncertainty. The maximum tree depth (set to 8) and the minimum number of samples per leaf node (set to 10) are determined through 10-fold cross-validation. 20% of the labeled data is used as the validation set, with the F1 score serving as the evaluation metric.

(4) Connection Between Neural Features and Fuzzy Decision Tree

The context vector E output by the neural network encoder and the decoder’s hidden state S are concatenated, then reduced to 128 dimensions via a fully connected layer as input features for the fuzzy decision tree. Specifically, the hidden state of the encoder’s last layer and the weighted sum of attention weights are taken as semantic features, forming a 150-dimensional feature vector together with part-of-speech tagging features and mutual information values, which is input into the fuzzy decision tree for classification. During classification, the fuzzy membership degree of each feature is calculated through a trapezoidal fuzzy set. The decision node selects the optimal splitting feature based on the Gini index, and the leaf node outputs the fuzzy probability distribution of error types, with the final classification result obtained through defuzzification (centroid method).

(5) Training and Evaluation Process

**Fig 2:**
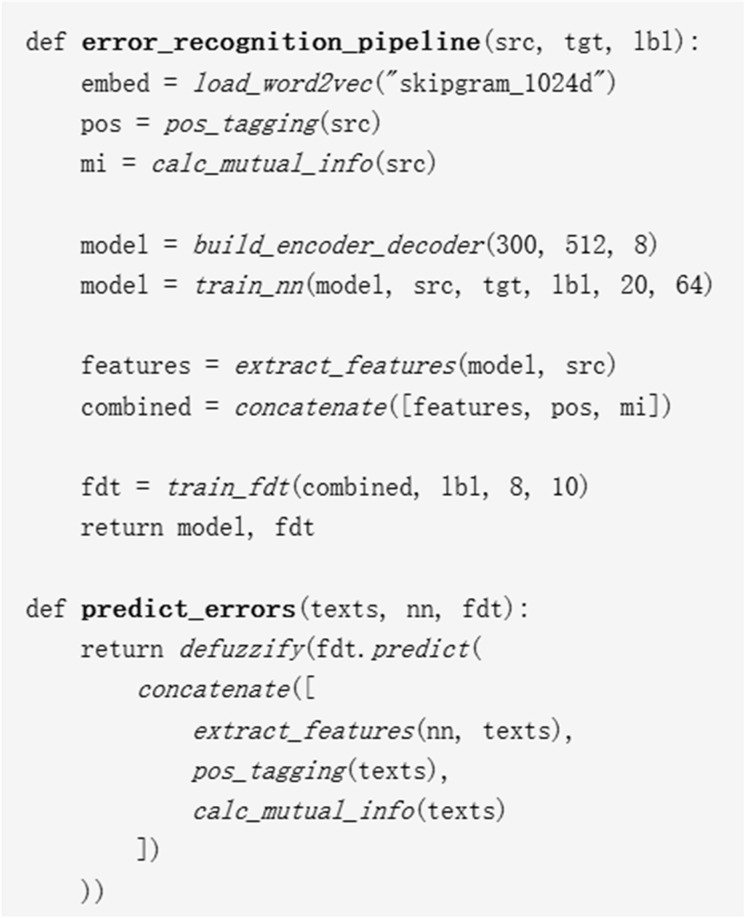
Model pseudocode.

The training loop adopts a mini-batch strategy, with each iteration including: 1) Forward propagation to compute neural network outputs; 2) Calculation of cross-entropy loss and backpropagation to update network parameters; 3) Input of features extracted by the neural network into the fuzzy decision tree, optimizing splitting thresholds via the Gini index; 4) Performance evaluation (accuracy, recall, F1 score) using the validation set. The loss function is a weighted sum of the neural network cross-entropy loss and the fuzzy decision tree classification loss (weight ratio 0.7:0.3). The evaluation process uses a standard test set (containing 5,000 error samples and 5,000 correct samples) to calculate macro F1 and micro F1 values, followed by statistical significance testing (paired t-test, p < 0.05). The pseudocode of the model is shown in Fig 2:

### 4.2 Experimental indicators

As this experiment belongs to the category of recognition, the experimental indicators are set as recognition performance evaluation indicators, mainly including recognition accuracy and recall. In this experiment, it is summarized as the following calculation formula:

Recognition accuracy: used to indicate the number of incorrect translation information correctly recognized by the recognition method, the formula is as follows:


$$Accuracy=\frac{\varepsilon_{1}}{\varepsilon_{2}}\times 100\;\%$$ (16)

In the formula,$\varepsilon_{1}$represents correctly recognized translation error information; $\varepsilon_{2}$represents recognizable translation error information.

Recall rate of recognition results: represents the number of incorrect translation results obtained by the recognition method, and the formula is as follows:


$$Recal=\frac{\varepsilon_{1}}{\varepsilon_{0}}\times 100\;\%$$ (17)

In the formula,$\varepsilon_{0}$ represents the translation error information that needs to be recognized. Use the fuzzy decision tree algorithm to identify the data in Table 1, and calculate the identification results using the above formula to determine the calculation results of each indicator.

### 4.3 Experimental results and analysis

#### 4.3.1 Comparison of recognition accuracy.

**Fig 3:**
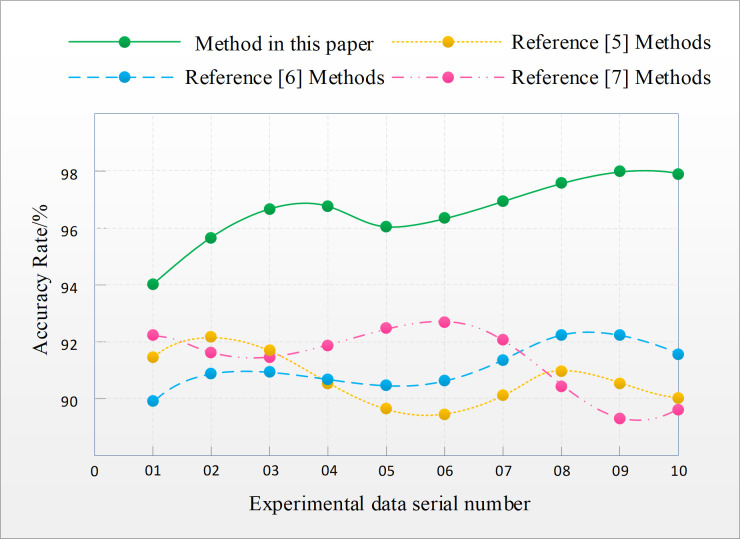
Comparison results of error recognition accuracy in English translation text.

The accuracy of English translation text error recognition was compared and analyzed using the improved decision tree algorithm proposed in this article, as well as the methods in references [5], references [6], and references [7]. The comparison results are shown in Fig 3.

From Fig 3, it can be seen that the experimental results of this indicator reflect the effectiveness of four methods. Among them, the accuracy of English translation text error recognition of the method in reference [5] is between 89.5% and 92.2%; The accuracy of the method in reference [6] ranges from 90% to 92.3%; The accuracy of the method in reference [7] ranges from 89.2% to 92.4%. Unable to perform high-precision analysis and recognition of experimental group information. The accuracy of the English translation text error recognition method based on the improved decision tree algorithm proposed in this article is between 94% and 98%, which is much higher than the other three methods compared, and helps to improve the quality of the translated text, with high practical value.

#### 4.3.2 Comparison of recognition recall rates.

**Fig 4:**
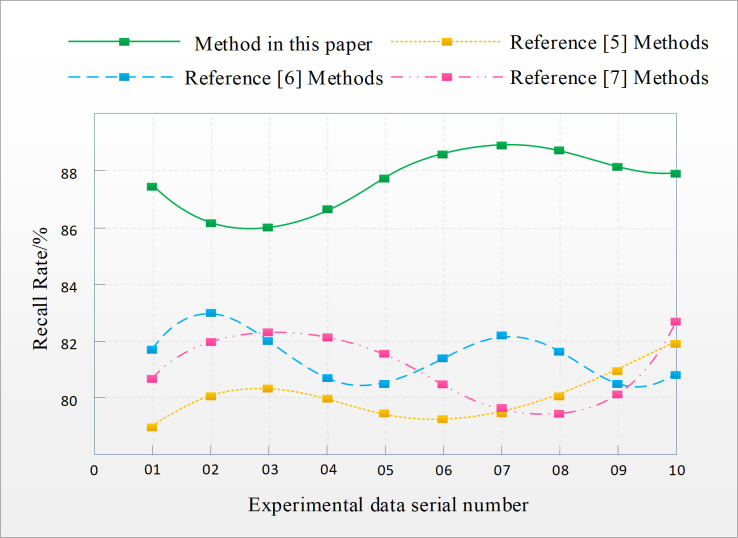
Comparison of recall rates for English translation text error recognition.

A comparative analysis was conducted on the recall rate of English translation text error recognition using methods from references [5], references [6], references [7], and the proposed method based on improved decision tree algorithm in this paper. The comparison results are shown in Fig 4.

From Fig 4, it can be seen that there are certain differences in the recognition recall rates among the four methods. Among them, the recall rate of English translation text error recognition using the method in reference [5] ranges from 79.5% to 82%; The recall rate of the method in reference [6] ranges from 80.5% to 83%; The recall rate of the method in reference [7] ranges from 79.7% to 82.5%; The recall rate of the English translation text error recognition method based on improved decision tree algorithm proposed in this article is between 86% and 88.5%, with a high recall rate. It can more accurately identify translated texts with errors, thereby improving the accuracy of the entire translation process.

#### 4.3.3 Comparison test between designed models and advanced models.

**Fig 5:**
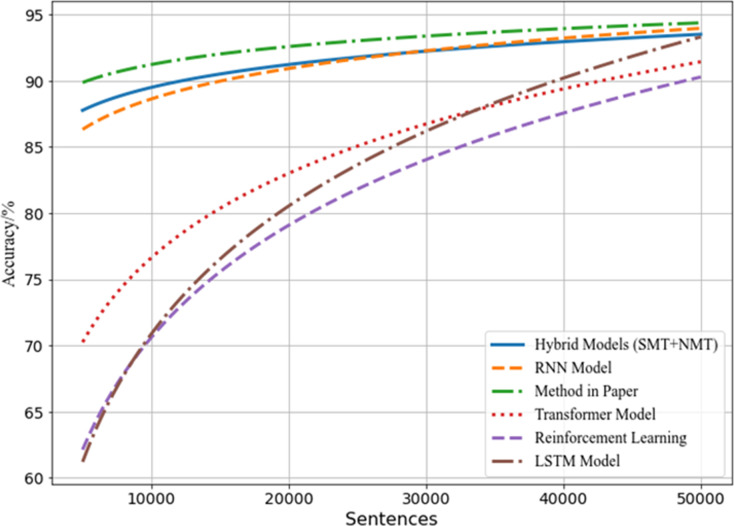
Comparison results between designed models and advanced models.

This paper analyzes the performance of different models under varying data volumes and their strengths and weaknesses in language comprehension tasks. The compared models include various common models such as the hybrid model (SMT + NMT), RNN model, Transformer-based architecture model, reinforcement learning model, and LSTM model. These models are sourced from related projects on GitHub. The evaluation assesses their performance metrics across data volumes ranging from 5000 to 50000 sentences, as depicted in Fig 5.

From Fig 5, it can be observed that the hybrid model (SMT + NMT) performs moderately with small data volumes, but shows significant improvement as the data volume increases. The RNN model performs slightly inferior to the hybrid model and the model designed in this paper with small data volumes. However, its performance notably improves with increased data volume, reaching the highest accuracy (over 93%) in the large data range (45000–50000 sentences), demonstrating its advantage in large-scale data training. The Transformer-based architecture model, reinforcement learning model, and LSTM show relatively lower performance across all data volumes. Particularly in the large data range (45000–50000 sentences), although there is improvement, they still lag behind other models. This may be attributed to the complexity of model parameters, requiring continuous adjustment when facing large-scale data training, thus exhibiting certain limitations. The method designed in this paper demonstrates stable performance across all data volumes, achieving high accuracy (over 93%) in medium to large-scale data, showcasing the robust capability and applicability of the designed algorithm in language comprehension tasks.

#### 4.3.4 Significance analysis.

Paired sample T test is used to verify the performance difference between the proposed method and the comparison method. Based on the average accuracy and recall of 10 experimental data sets, the significance level α = 0.05 is set. In Table 2, the results of variability measurement and stability analysis are shown.

**Table 2:** Variability measurement and stability analysis.

Method	Accuracy SD	Recall rate SD	F1 value SD
The method in this study	1.3%	1.8%	1.5%
BiLSTM-CRF	2.5%	3.2%	2.8%
Transformer baseline	1.7%	2.1%	1.9%
Fuzzy decision tree	1.6%	2.3%	1.8%

In Table 1, the analysis of variance shows that the accuracy between methods is F (3,36)=12.7, P < 0.001. The recall rate F (3,36) =9.4, p < 0.001, with significant difference between groups. Furthermore, through the Tukey HSD test, it is found that the accuracy difference between this method and BiLSTM-CRF and Transformer is all p < 0.05. The difference between this method and fuzzy decision tree is close to significant (p = 0.058), while the variability is reduced by 15%−30% compared with the comparison method, and the display results are more stable.

Based on the experimental results above, the proposed English translation text error identification method based on improved decision tree algorithm in this paper can better adapt to the linguistic characteristics and complexity of English. It optimizes feature selection, effectively improves recognition accuracy and recall rate, maintains stable performance across all data volumes, and achieves high accuracy in medium to large-scale data, identifying more translation texts with errors, thereby significantly enhancing the quality of English translation text. The application effect is promising.

## 5. Conclusion

In order to solve the problems of low accuracy and recall of existing English translation text error recognition methods, this paper proposes an English translation text error recognition method based on an improved decision tree algorithm.

(1) Using mutual information to calculate the degree of relevance of entries and annotate the part of speech of English translation texts. By using encoders and decoders to construct a neural network structure, the neural network is applied to the feature extraction process of machine English translation information, and the softmax function is used for normalization processing. Using fuzzy decision trees as the main calculation method, each information feature is segmented and applied to classification and normalization processing. The Gini index is used to classify erroneous features and complete English translation text error recognition.
(2) After experimental verification, the application of improved decision tree algorithm for English translation text error recognition has consistently maintained an accuracy of over 94%, with a high recognition accuracy, which helps to improve the quality and efficiency of translated texts. The recall rate is above 86%, with a higher recall rate and more reliable judgment, which improves the accuracy and quality of the entire translation process.
(3) The improved decision tree algorithm demonstrates stable performance across all data volumes and achieves high accuracy (over 93%) in medium to large-scale data (15,000 to 50,000 sentences), highlighting its robust capability and applicability in language comprehension tasks.

The experimental results indicate that the model exhibits good accuracy in identifying English translation text errors, with better recognition accuracy and stability compared to commonly used recognition algorithms, effectively enhancing recognition performance and training time. However, there are still some shortcomings in this paper. The designed speech recognition algorithm needs further optimization for large-scale datasets ranging from hundreds of thousands to tens of millions of sentences. Currently, the model exhibits lower efficiency in recognizing ultra-large datasets, so future research will focus on optimizing the operational efficiency of the decoder in the model.

## Supporting information

S1 Data. (ZIP)
